# *TMTC2* variant associated with sensorineural hearing loss and auditory neuropathy spectrum disorder in a family dyad

**DOI:** 10.1002/mgg3.397

**Published:** 2018-04-19

**Authors:** Hector Guillen‐Ahlers, Christy B. Erbe, Frédéric D. Chevalier, Maria J. Montoya, Kip D. Zimmerman, Carl D. Langefeld, Michael Olivier, Christina L. Runge

**Affiliations:** ^1^ Department of Genetics Texas Biomedical Research Institute San Antonio TX USA; ^2^ Department of Otolaryngology and Communication Sciences Medical College of Wisconsin Milwaukee WI USA; ^3^ Department of Biostatistical Sciences Wake Forest University School of Medicine Winston‐Salem NC USA; ^4^ Present address: Department of Internal Medicine Section of Molecular Medicine Wake Forest University School of Medicine Winston‐Salem NC USA

**Keywords:** auditory neuropathy spectrum disorder, cochlear implant, genetics, sensorineural hearing loss, TMTC2

## Abstract

**Background:**

Sensorineural hearing loss (SNHL) is a common form of hearing loss that can be inherited or triggered by environmental insults; auditory neuropathy spectrum disorder (ANSD) is a SNHL subtype with unique diagnostic criteria. The genetic factors associated with these impairments are vast and diverse, but causal genetic factors are rarely characterized.

**Methods:**

A family dyad, both cochlear implant recipients, presented with a hearing history of bilateral, progressive SNHL, and ANSD. Whole‐exome sequencing was performed to identify coding sequence variants shared by both family members, and screened against genes relevant to hearing loss and variants known to be associated with SNHL and ANSD.

**Results:**

Both family members are successful cochlear implant users, demonstrating effective auditory nerve stimulation with their devices. Genetic analyses revealed a mutation (rs35725509) in the *TMTC2* gene, which has been reported previously as a likely genetic cause of SNHL in another family of Northern European descent.

**Conclusion:**

This study represents the first confirmation of the rs35725509 variant in an independent family as a likely cause for the complex hearing loss phenotype (SNHL and ANSD) observed in this family dyad.

## 1 BACKGROUND

Hearing loss (HL) is a common sensory disorder often identified at birth (1 in 1,000 newborns), with the incidence increasing to 0.5% through childhood (Morton, 1991). With advances in genetic testing for SNHL, clinical platforms that screen for known SNHL mutations, such as the OtoGenome™ test (http://personalizedmedicine.partners.org/Laboratory-For-Molecular-Medicine/Tests/Hearing-Loss/OtoGenome.aspx) help identify the genetic cause for developing hearing loss, but only a limited number of mutations causing SNHL are known to date.

Exome sequencing is a valuable approach in the clinic for diseases such as hereditary hearing loss, which can be caused by a large number of mutations in a wide range of different genes (hereditaryhearingloss.org). In conjunction with phenotypical and clinical assessments, exome sequencing should be taken into consideration during the patient assessment and development of intervention plans when clinical screening tools do not reveal the genetic cause, given the effects of different etiologies in responses to treatment options such as hearing aids and cochlear implants (Eppsteiner et al., 2012).

For this study, two members of a family of Northern European descent (mother and son) with SNHL were analyzed to identify the genetic cause of SNHL shared by both individuals. The son was subsequently diagnosed with ANSD, a sub‐type of SNHL. Exome sequencing of both affected family members was used to identify the likely causal variant(s) for the hearing impairment.

## 2 METHODS

### 2.1 Study design and participants

Participants were mother (S61) and son (S60) cochlear implant recipients. Both were unilaterally implanted with Cochlear™ Freedom^®^ devices in the left ear. They were of Northern European descent, and provided DNA samples and permission to access clinical records.

The procedures followed were in accordance with the ethical standards of the responsible committee on human experimentation and with the Helsinki Declaration (World Medical Association, 2013). All procedures were approved by the local IRB.

### 2.2 Clinical assessment

Clinical data included family history, preoperative audiograms, pre and postoperative speech perception scores, and electrically evoked compound action potentials (ECAPs). Speech perception tests were all presented using recorded materials at 60 dB SPL (Firszt et al., 2004), including the Hearing in Noise Test (HINT) and HINT Children's Version (HINT C) (Nilsson, Soli, & Gelnett, 1996; Nilsson, Soli, & Sullivan, 1994) presented in quiet (Q) and in +8 signal‐to‐noise ratio (SNR) speech‐weighted noise (N), AzBio presented in quiet and in +8 SNR multitalker babble noise (N) (Spahr et al., 2012), Phonetically Balanced Kindergarten (PBK) word list (Haskins, 1949) and Consonant‐Nucleus‐Consonant (CNC) word list (Peterson & Lehiste, 1962).

### 2.3 DNA sample collection

Saliva samples were obtained and DNA extracted using Oragene Collection Kits and Oragene prepIT‐LP2 reagent (DNA Genotek, ON, Canada).

### 2.4 Exome sequencing

DNA libraries were generated using the Nextera Rapid Capture Exome Enrichment Kit (Illumina), following the manufacturer's guidelines and quantified by qPCR. Libraries were sequenced on an Illumina HiSeq 2500 Sequencing System using a 2 × 100 bp paired‐end run.

### 2.5 Sequence data processing and variant identification

The sequencing data were aligned against the human reference genome (GRCh37) using BWA (v.07.12) (Li & Durbin, 2010) and SAMtools (v.1.2) (Li et al., 2009). The base quality score recalibration was performed using the BaseRecalibrator module of GATK (v1.4‐37) (McKenna et al., 2010) and the dbSNP build 142. Variant calling was performed using the UnifiedGenotyper module of GATK (v1.4‐37) (McKenna et al., 2010). Bait intersection, read depth, and capture efficiency analyses were performed using BEDTools (v2.21.0) (Quinlan & Hall, 2010). The variant calling was filtered using a minimum GQ score of 90 and a minimum read depth of 6. Only variants present in both family members were included in further analysis.

### 2.6 Copy number analysis

For each sample, copy number variants were characterized through the application of Control‐FREEC (Boeva et al., 2012). The genome was divided into small adjacent regions using a sliding window approach and read count profiles were computed for each region and normalized to adjust for GC‐content. A penalized regression approach, LASSO was used to model the copy number ratios and positions with nonzero coefficients were considered as change points (Berk, 2008). Lastly, a Kolmogorov‐Smirnov test was implemented to assess the false‐positive rate of each detected CNA. Loss‐specific CNAs were retained and annotated via ANNOVAR (Wang, Li, & Hakonarson, 2010) to produce lists of the genes in these regions.

## 3 RESULTS

### 3.1 Phenotypic information

**Figure 1:**
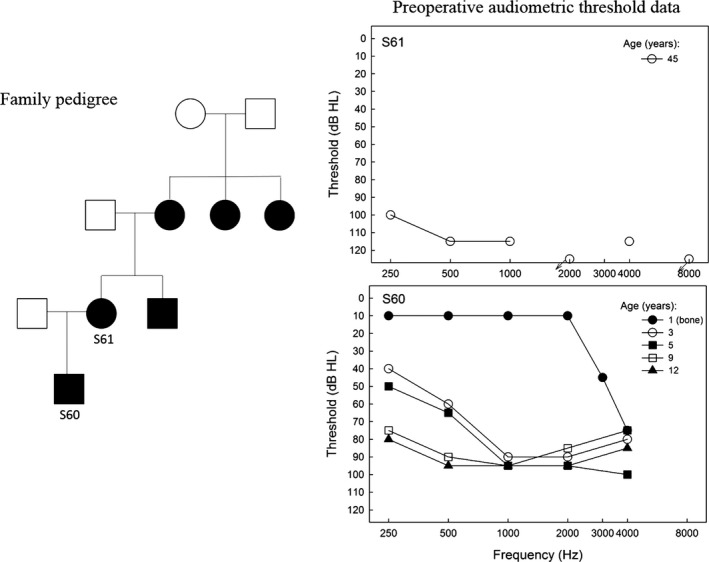
Family pedigree and preoperative audiometric thresholds (upper graph S61, lower graph S60)

A history of SNHL was reported in 6 members of the family: S60, S61, S60s uncle, grandmother, and great aunts (Figure 1). S60 was diagnosed with bilateral ANSD at 12 years of age (abnormal auditory brainstem responses (ABR) with present otoacoustic emissions). The mother was diagnosed with SNHL, but did not undergo evaluation for ANSD. Both S60 and S61 had a history of childhood‐onset (1 and 5 years of age, respectively) bilateral, symmetric hearing loss, progressing to severe‐to‐profound loss (3 and 43 years of age) warranting cochlear implantation (at 13 and 46 years of age).

Preimplant audiometric thresholds for both subjects, and longitudinal data for the son, are shown in Figure 1 (right ear only is shown for simplicity; hearing losses were symmetric between right and left ears). Preimplant, S61 had thresholds in the profound hearing loss range. Hearing loss progression was observed in the longitudinal data for S60, starting with high‐frequency hearing loss at 1 year of age, with progression to severe‐to‐profound hearing loss by age 9.

Cochlear implant outcomes indicated effective neural stimulation and speech perception for both subjects. Both utilize a bimodal listening configuration (cochlear implant in the left ear, hearing aid in the right ear). Electrically evoked compound action potential (ECAP) thresholds measured by Neural Response Telemetry (tNRT) are shown in Table 1, along with average Nucleus Freedom tNRTs adapted from van Dijk et al. (2007) for comparison. Both subjects had lower ECAP thresholds compared to cochlear implant users with comparable devices, indicating effective stimulation of the auditory nerve.

**Table 1:** Electrically evoked compound action potential thresholds and speech perception scores

Electrode	S61 tNRT	S60 tNRT	Avg tNRT (van Dijk et al., 2007)
20	167	152	171
16	146	146	165
11	161	167	182
1	173	158	182

	HINT (C) Q		HINT C N		AzBio Q		AzBio N		PBK		CNC	
	S61	S60	S61	S60	S61	S60	S61	S60	S61	S60	S61	S60
Pre‐I HA	0	85	a	31	a	a	a	a	a	36	0	a
6 month Cl + HA	a	91	a	52	a	a	a	a	a	44	a	70
9 year Cl alone	a	a	a	a	44	93	a	53	a	a	40	70
9 year Cl + HA	a	a	a	a	45	98	a	62	a	a	52	78

CI, cochlear implant; CI + HA, bimodal cochlear implant and hearing aid; m, months postimplant; Pre‐I HA, preimplant; tNRT, neural response telemetry threshold; y, year postimplant.

All values represent percent correct.

a Not tested.

Pre and postimplant speech perception scores for S61 and S60 are shown in Table 1. Both participants had higher scores with cochlear implant use compared to preoperative performance, and also indicated bimodal benefit for both.

### 3.2 *TMTC2* gene variant rs35725509 as the likely cause for hearing loss

Coding variants across the entire genome were identified with percent coverage, mean, and median read depths for S61 of 99%, 172.6%, and 120%, respectively, and 86%, 23.22%, and 11% for S60. A total of 7,441 sequence variants were identified, 1,921 (25.8%) of which were nonsynonymous present in both individuals. Twenty‐five of these variants were located in the coding sequence of 17 of 146 previously identified hearing loss genes (Table 2). Special attention was given to mitochondrial DNA, since the family pedigree (Figure 1) shows that in two generations, the trait has been passed through the maternal side, but no shared variants were detected in the mitochondria.

**Table 2:** Nonsynonymous variants coinciding with hearing loss‐related genes

SNP	Allele	EA AF (%)	Conditions Mentioned in the Literature	Gene	S60				S61			
					GT	*g* $\overset{¯}{x}$DP	*g* M DP	*g* CVG	GT	*g* $\overset{¯}{x}$DP	*g* M DP	*g* CVG
rs36009281	G	8.34	–	BDP1	0/1	18.7	16	99%	1/1	117.6	112	100%
rs3761967	A	48.19	–	BDP1	0/1	18.7	16	99%	1/1	117.6	112	100%
rs1961760	A	48.34	Recessive NHL (Chishti et al., 2008)	BDP1	0/1	18.7	16	99%	1/1	117.6	112	100%
rs2028574	T	39.58	Progressive HL (Modamio‐Høybjør et al., 2007)	CCDC50	0/1	28.1	25	78%	1/1	206.8	186	80%
rs293813	T	46.10	Progressive HL (Modamio‐Høybjør et al., 2007)	CCDC50	0/1	28.1	25	78%	1/1	206.8	186	80%
rs1045644	G	37.04	Ménière's Disease (Vrabec, Liu, Li, & Leal, 2008)	COCH	0/1	26.4	27	93%	0/1	226.0	225	100%
rs34392760	A	5.30	–	COL2A1	0/1	18.7	15	96%	0/1	415.2	400	100%
rs2229813	T	42.19	–	COL4A4	0/1	27.9	22	98%	0/1	215.6	204	100%
rs592121	G	37.76	–	COL9A1	0/1	744.2	35	100%	0/1	285.9	285	100%
rs2274305	T	65.08	–	DCDC2	0/1	25.3	20	95%	1/1	123.0	109	100%
rs25640	A	46.00	–	HSD17B4	0/1	36.7	34	97%	1/1	234.6	228	100%
rs11205	G	40.91	–	HSD17B4	0/1	36.7	34	97%	0/1	234.6	228	100%
rs1377016	A	31.20^a^	–	LOXHD1	1/1	27.6	17	96%	1/1	257.9	220	99%
rs3824700	A	44.99	–	MYO3A	0/1	39.2	34	100%	1/1	292.8	278	100%
rs3824699	A	68.38	–	MYO3A	1/1	39.2	34	100%	1/1	292.8	278	100%
rs3758449	A	45.08	–	MYO3A	0/1	39.2	34	100%	1/1	292.8	278	100%
rs3740231	T	42.95	–	MYO3A	0/1	39.2	34	100%	1/1	292.8	278	100%
rs10825269	T	12.80	–	PCDH15	0/1	58.6	39	94%	0/1	254.5	236	94%
rs2295769	C	29.93	–	SERPINB6	0/1	31.2	24	93%	0/1	242.3	223	93%
rs272893	T	61.52	–	SLC22A4	0/1	26.5	20	100%	0/1	271.7	285	100%
rs1050152	T	41.94	–	SLC22A4	0/1	39.2	34	100%	0/1	271.7	285	100%
rs72768728	G	1.14	–	TBC1D24	0/1	7.0	7	72%	0/1	280.7	264	91%
rs35725509	A	**0.77**	NSHL (Runge et al., 2016)	TMTC2	0/1	11.4	9	98%	0/1	114.5	94	100%
rs1061494	C	43.77	–	TNC	0/1	13.7	10	89%	0/1	134.2	112	100%
rs1801212	G	72.37	Wolfram Syndrome (Aloi et al., 2012)	WFS1	1/1	15.8	8	93%	1/1	245.9	166	100%

AF, allele frequency; EA, European American; *g* CVG, gene coverage; *g* $\overset{¯}{x}$ /M DP, gene mean/median read depth; GT, genotype (0 and 1 refer to the reference and derived alleles, respectively); HL, hearing loss; NHL, nonsyndromic HL; NSHL, nonsyndromic sensorineural HL; SNP, single‐nucleotide polymorphism.

Allele frequencies (Exome Variant Server, NHLBI GO Exome Sequencing Project, Seattle, WA (evs.gs.washington.edu/EVS) [09/2017]) and reported disease associations are shown for each variant. Genotype, read depth, and coverage for each sample and gene are shown on the right. Allele frequencies below 1% are highlighted in bold.

a Not shown as a variant at EVS.

Since hereditary hearing loss is relatively rare in the general population, variants with minor allele frequencies (MAF) above 1% are considered unlikely to be causal mutations, otherwise this hearing impairment would be more prevalent. Only rs35725509 in the *TMTC2* gene showed a MAF below 1% in 2,203 individuals of European American (EA) ancestry (NHLBI GO Exome Sequencing Project, http://evs.gs.washington.edu/EVS/). A far more permissive EA MAF threshold of 5% would only add rs34392760 and rs72768728 in the *COL2A1* and *TBC1D24* genes, respectively. Apart from their relatively high MAF values, these two additional variants are predicted to be benign in their impact on normal gene function, and have not previously been reported as likely mutations causing SNHL. In contrast, rs35725509 in the *TMTC2* gene was recently identified in another unrelated family with SNHL (Runge et al., 2016). The rs35725509 variant was the only mutation (nonsynonymous or synonymous) found in the coding region of the *TMTC2* gene in S60 or S61.

Normalized copy number profiles failed to reveal any shared changes, between both family members, within hearing loss‐annotated regions.

## 4 DISCUSSION

*TMTC2* is a gene that, outside this and one other study (Runge et al., 2016), had not been previously implicated in hearing loss. In both cases, a strong association of the rs35725509 variant to the nonsyndromic SNHL phenotype was uncovered. Lack of mutations in other well‐established hearing loss‐related genes further supported rs35725509 as the likeliest causal variant, based on the inheritance pattern of the mutation, and elevated MAFs observed in the EA population for the other remaining potential candidates. *TMTC2* is expressed in many tissues (Wu, MacLeod, & Su, 2013) including the inner ear (Runge et al., 2016). While the specific function(s) of *TMTC2* within the auditory system remain unknown, the protein is likely to affect ion currents and membrane potential in inner ear cells, based on reported functions in other cell types (Sunryd et al., 2014).

Our sequence analysis of individuals S60 and S61 did not uncover any nonsynonymous variants in genes already known to be associated with ANSD such as autosomal recessive *OTOF* (Yasunaga et al., 1999) and *PJVK* (Delmaghani et al., 2006), and autosomal dominant *AUNA1* (Kim et al., 2004), leaving rs35725509 in the *TMTC2* gene as the likely causal mutation. Even the recently proposed *AUNA2* (Lang‐Roth et al., 2017), encompassing 1.7 and 3 Mb on regions 12q24 and 13q34, respectively, did not add any likely candidates. However, our sequencing efforts did uncover five additional nonsynonymous variants in other genes previously reported to contain mutations associated with hearing impairments (Table 2). All these variants have MAF values above 25%, likely disqualifying them as causal mutations in this relatively rare (monogenic) form of SNHL.

Finally, variant rs72768728 in the *TBC1D24* gene, which is relatively rare (EA MAF 1.1%), is predicted to be benign and has never been reported before to cause HL‐related phenotypes.

The lack of shared mitochondrial mutations likely eliminates the possibility to venture about maternal transmission through this path. Additionally, the copy number profiles obtained in our analysis did not reveal any overlap in relevant HL‐regions for it to be considered as an alternative explanation for the observed phenotypes.

Phenotype characteristics and effective intervention outcomes for S60 and S61 were similar to those previously reported for an unrelated family of Northern European descent who also harbored the *TMTC2* rs35725509 variant (Runge et al., 2016). Objective ECAP measures and speech perception abilities indicated effective stimulation of the auditory system with cochlear implants, and are consistent with postlingual hearing loss onset. In contrast to the previously reported family, S60 was diagnosed with ANSD. Recovery of synchronized neural responses and speech perception with cochlear implantation in ANSD is well documented (Shallop et al., 2005; Peterson et al., 2003; Breneman et al., 2012; Berlin et al., 2010) including bimodal benefit (Runge et al., 2011). Differentiating between pre or postsynaptic site of lesion in ANSD is difficult to discern with current clinical assessment tools. ANSD‐associated genes *OTOF* and *AUNA1* are expressed in the cochlea, indicating presynaptic site of lesion, and *PJVK* is expressed in the auditory nerve, indicating postsynaptic site of lesion (Yasunaga et al., 1999; Delmaghani et al., 2006; Schoen et al., 2010). In cases of ANSD with significant neural pathology, such as Friedreich's Ataxia, patients experience inconsistent benefit from cochlear implantation (Frewin et al., 2013; Miyamoto et al., 1999). Our previous study identified *TMTC2* expression in the human cochlea (Runge et al., 2016). While speculative, the expression data, phenotypes, and highly effective responses to cochlear implantation in the unrelated family of Northern European descent and S60 and S61 suggest a presynaptic site of lesion for the *TMTC2* rs35725509 variant. The current findings suggest that *TMTC2* rs35725509 affects a range of auditory phenotypes, leading to bilateral SNHL, and possibly ANSD. *TMTC2* is a novel gene that should be considered when searching for potential causal mutations in inherited SNHL and ANSD.

## CONFLICT OF INTEREST

None declared.
